# Molecular and Microscopic-Based Characterization of *Plasmodium* spp. in Fars and Hormozgan Provinces, South of Iran

**DOI:** 10.1155/2014/935469

**Published:** 2014-02-06

**Authors:** Tahereh Mohammadzadeh, Gholamreza Hatam, Mohsen Kalantari, Bahador Sarkari, Mohammad Hosein Motazedian, Seyed Mahmoud Sadjjadi, Reza Safari

**Affiliations:** ^1^Department of Parasitology and Mycology, School of Medicine, Shiraz University of Medical Sciences, Shiraz, Iran; ^2^Department of Parasitology and Mycology, School of Medicine, Baqiyatallah University of Medical Sciences, Tehran, Iran; ^3^Basic Sciences in Infectious Diseases Research Center, School of Medicine, Shiraz University of Medical Sciences, Shiraz, Iran; ^4^Hormozgan Health Centre, Hormozgan University of Medical Sciences, Bandar Abbas, Iran

## Abstract

Despite malaria control programs in recent years, malaria transmission has not been eliminated in Iran. Molecular techniques including PCR, which has proved more sensitive and specific than microscopic examination methods, help to detect infection in low levels of parasitemia and mixed infections. Main our objectives were setting up nested PCR for detection of malaria and evaluating PCR based on plasmodia DNA from blood smears in Fars province, the comparison of this method with traditional microscopy and also evaluate the data in comparison with its neighboring province, Hormozgan. A total of 149 malaria positive samples including 116, 19, and 14 samples from Shiraz, Jask, and Lengeh ports were utilized in this study, respectively. Blood slides were prepared for microscopic observation. DNA from thin smears was extracted and nested PCR was analyzed using rPLU5 and rPLU6 for genus specification, rFAL1, rFAL2, and rVIV1, rVIV2 for *P. falciparum* and *P. vivax* detection, respectively. The results showed that 126 (84.6%), 16 (10.7%), and 7 (4.7%) out of 149 cases were positive for *P. vivax*, *P. falciparum,* and mixed infections, respectively, by microscopy. The PCR indicated that 95 (63.7%), 15 (10.1%), and 22 (14.8%) cases were infected with *P. vivax*, *P. falciparum,* and mixed mentioned species, respectively, and 17 (11.4%) cases were uninfected. Our results confirmed the considerable sensitivity of nested PCR for detection of the mixed infections. Simultaneous application of PCR even based on microscopy slides can facilitate access to the highest level of confidence in malaria researches.

## 1. Introduction

Despite malaria control programs in recent years, malaria is one of the most important infectious parasitic diseases in the world. According to the latest World Health Organization report, 216 million episodes of malaria and 655,000 malaria deaths were estimated in 2010 [1]. Although Islamic Republic of Iran has been in the elimination phase since 2008 and 2010, respectively [2], malaria is still present in some provinces. Hormozgan, Kerman, and Sistan-Baluchestan provinces located in the south and south-eastern part of Iran are the most important areas of malaria transmission, respectively [3]. Hormozgan and Kerman are Fars's neighboring provinces and due to the communication and travels between these regions, especially migration of foreign workers can increase susceptibility of Fars to this disease. Based on a 1999 report, the highest incidence of the disease was reported in Lar City (64/100000 people) and the City of Neyriz (57/100000 people) [4].

Gold standard diagnostic method of malaria is based on microscopic examination. Since the microscopic observation of malaria parasites is difficult in low parasitemia blood smears [5, 6] and it leads to some problems in the output of epidemiological studies; using molecular techniques such as PCR, which has proved more sensitive and specific than microscopic evaluation, can help to detect infection in patients with low level of parasites and mixed infections [7–11].

Furthermore, molecular characterization of parasites can be used for any survey on drug resistance, pathogenicity, and molecular epidemiology. Many studies have been done in recent years in our country. Detection of malaria was done by nested PCR and microscopy in Chabahar [12, 13]. Molecular epidemiology of malaria was evaluated in three endemic areas (Sistan-Baluchestan, Kerman, and Hormozgan) and the results were compared by microscopy [8]. Direct PCR and microscopy were applied for detection of *P. falciparum* in people with malaria-like symptoms and fever in Zahedan [14].

Nested PCR has also been utilized on DNA from historical malaria negative samples in Sistan- Baluchestan [9]. Some Iranian scientists have tried MSP gene [15, 16]. Zakeri et al. have evaluated malaria in three countries (Iran, Afghanistan and Pakistan) by molecular and microscopic methods [7]. Metacaspase 1 gene in *Plasmodium vivax* has characterized for some isolates from south of Iran [17].

Molecular method and microscopy were compared with serologic method in a survey for determination of asymptomatic malaria cases [18].

Unfortunately, there are no important reports about molecular work on malaria in Fars province.

Regarding the importance of the incidence and transmission of dynamics by epidemiological studies in malaria control [19], in the present study, our main objectives were setting up nested PCR to detect malaria and evaluate PCR based on plasmodia retrieved from blood smear in Fars province, south of Iran. We also aimed to compare this method with traditional microscopy and also evaluate the data in comparison with its neighboring province, Hormozgan.

## 2. Materials and Methods

### 2.1. Study Regions

Fars province is in the southern center of the country and its center is Shiraz. It has an area of 122,400 km². According to the report of 2011 National Population and Housing Census of Iran, the Fars's population have exceeded 4500000 (4596658). There are three distinct climatic regions in the Fars Province: mountainous area of the north and northwest with moderate cold winters and mild summers, central regions with relatively rainy mild winters and hot dry summers and south and southeast parts which have moderate winters with very hot summers [20].

Kerman is located in the east of Fars and Hormozgan is south-eastern and southern neighbor of Fars which both are endemic areas for malaria [3].

Hormozgan province is in the south of the country, facing Oman and United Arab Emirates. Its area is 70,697 km^2^ and its center is Bandar Abbas. The province has 14 islands located in the Persian Gulf and 1,000 km (620 mi) of coast line [20].

According to the report of 2011 National Population and Housing Census of Iran, the population of Fars have exceeded 1500000 (1578183). The province is primarily mountainous, consisting of the southern tip of the Zagros Range. The province experiences a very hot and humid climate, with temperatures sometimes exceeding 120°F (49°C) in summers. Kerman and Sistan-Baluchestan are located in the north and east of Hormozgan, respectively, and are both endemic areas of malaria such as their neighbor Hormozgan [20].

During 2008-2009, a total of 149 malaria positive samples from different ethnic and racial groups were collected, transferred to Shiraz University of Medical Sciences, Parasitology Department (SUMSPD), and utilized in this study. From the total of 149 samples, 116 samples were from Shiraz (Valfajr Health Care Center), while 33 samples were from Hormozgan province (19 from Jask and 14 from Lengeh port).

Different data including age, gender, education level, nationality, lodging and clinical signs, history of the other diseases, and drug taken were recorded for each patient in a questionnaire.

### 2.2. Microscopic Examination

Blood samples were collected and thin slides were prepared for microscopic observation.

Sample film fixation and the Giemsa-staining were done in the three above-mentioned health centers. Microscopy examination was utilized by a well-trained malariologist in health centers and a parasitologist.

### 2.3. Molecular Assay

PCR was performed on all of the stained microscopic slides after collecting all of the samples. To do that, the immersion oil was wiped off with a paper tissue from each slide. The thin smear was then scraped off the slide, so the total DNA in the smear was extracted by digestion, in a 1.5 mL microcentrifuge tube, with 200 µL lysis buffer (1 mM ethylene diamine tetra acetic acid, 50 mM Tris-HCl (pH 7.6), 1% Tween 20 (v/v)) containing 5 µL of a proteinase K solution. The tube was incubated overnight, at 37°C, before 200 µL phenol/chloroform/isoamyl alcohol (25 : 24 : 1, by vol) was added to it. The tube was then shaken vigorously and centrifuged at 12000 rpm for 10 min. The resultant supernatant solution was mixed with 300 µL cold absolute ethanol. The precipitated DNA was centrifuged down (at 12000 rpm for 10 min), dried, dissolved in 100 mL deionized distilled water, and then stored at −20°C until it could be tested [21, 22].

In an initial amplification reaction, the primer pair rPLU5 (5′-CCT GTT GTT GCC TTA AAC TTC-3′) and rPLU6 (5′-TTA AAA TTG TTG CAG TTA AAACG-3′) were used [11].

The size of DNA target, amplified by these outer primers, was about 1200 bp. These primers were genus specific and can, therefore, amplify the target sequences from all four species of human malaria parasite (*P. falciparum*, *P. vivax*, *P. malariae*, and *P. ovale*) [23, 24].

The second reaction was performed for the specific detection of the above *Plasmodium spp*. using a set of two primer pairs; rFAL1 (5′-TTA AAC TGG TTT GGG AAA ACC AAA TAT ATT-3′) and rFAL2 (5′-ACA CAA TGA ACT CAA TCA TGA CTA CCC GTC-3′) for *P. falciparum* and rVIV1 (5′-CGC TTC TAG CTT AAT CCA CAT AAC TGA TAC-3′) and rVIV2 (5′-AAG GAA AGA AAG TCC TTA-3′) for *P. vivax* detection [24].

Positive control samples were obtained from Pasture Institute, Tehran, Iran. A negative control was also established for each serial of reactions. Each 20 *μ*L reaction mixture for the first amplifications contained 5 *μ*L of template DNA, 2 *μ*L of 10x PCR buffer (50 mM KCl, 10 mM Tris-HCl), 4 mM MgCl_2_, 200 *μ*M of each dNTPs, 0.4 units of Taq DNA Polymerase, and 250 nM of each primer (rPLU5 & rPLU6). The PCR conditions were as follows: 95°C for 5 min; 94°C for one min, 58°C for two min; extension at 72°C for two min; 25 cycles and final extension at 72°C for 7 min. About 2 *μ*L of the first amplification products was served as the DNA template for each of the 20 *μ*L of the second PCR amplification. The concentrations of the second primers and other constituents were identical to the first amplification, except that 0.3 unit of Taq DNA polymerase was used [9, 23].

PCR products was subjected to gel electrophoresis on a 1.5% agarose gel, stained with ethidium bromide, and visualized under UV light [25].

## 3. Results

One hundred forty-nine malaria patients were evaluated by nested PCR. One hundred sixteen samples were from Shiraz (Fars province) and all cases except one were male and most of them were immigrant foreigners (Afghan). Nineteen and fourteen samples were from Jask and Lengeh ports respectively. Most of those from Jask port and all patients from Lengeh port were Iranian and male.

### 3.1. Microscopic Examination

The results showed that 126 (84.6%), 16 (10.7%), and 7 (4.7%) out of 149 cases were positive for *P. vivax*, *P. falciparum*, and mixed infections, respectively, by microscopy. The results of Valfajr Healthcare Centre in Shiraz, Fars, showed that 97 (83.6%), 13 (11.2%), and 6 (5.2%) out of 116 Shiraz samples were positive for *P. vivax*, *P. falciparum* and mixed infections, respectively. Also, the results obtained from Jask and Lengeh Health Care Centers in Hormozgan showed that 18 (94.7%) and 11 (78.6%) were positive for *P. vivax*, 1 (5.3%) and 2 (14.3%) positive for *P. falciparum*, and 0 (0%) and 1 (7.1%) positive for mixed infections, respectively. Details of the results are displayed in Table 1.

### 3.2. Molecular Assay

**Figure 1:**
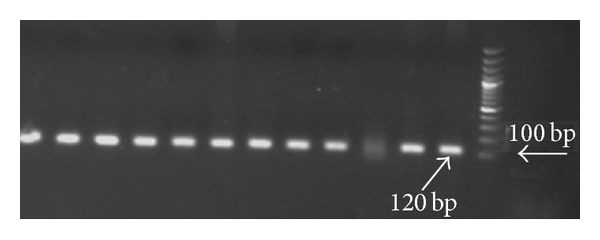
Agarose gel electrophoresis of nested PCR products from clinical specimens using species-specific oligonucleotide pairs for *P. vivax*.

**Figure 2:**
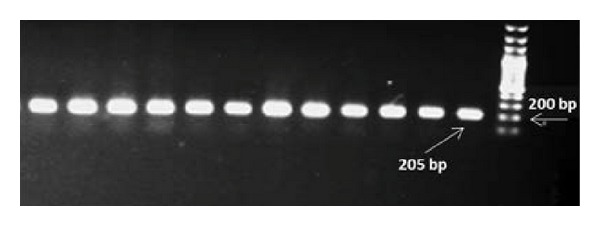
Agarose gel electrophoresis of nested PCR products from clinical specimens using species-specific oligonucleotide pairs for *P. falciparum*.

The nested PCR indicated that 95 (63.7%), 15 (10.1%), and 22 (14.8%) cases were infected with *P. vivax*, (Figure 1), *P. falciparum* (Figure 2), and mixed forms of both mentioned species, respectively, and 17 (11.4%) cases were uninfected. Our results revealed that 69 (59.5%), 13 (11.2%), and 21 (18.1%) out of 116 samples from Shiraz were positive for *P. vivax*, *P. falciparum*, *and* mixed infections, respectively, and 13 (11.2%) were also negative samples. In addition, the results obtained from Jask and Lengeh Health Care Centers showed that 17 (89.4%) and 9 (64.3%) are positive for *P. vivax*, 1 (5.3%) and 1 (7.1%) positive for *P. falciparum*, and 1 (5.3%) and 0 (0%) positive for mixed infections, respectively. None (0%) of the patients in Jask and 4 (28.6%) samples from Lengeh were negative.The results are shown in Table 1.

## 4. Discussion

According to WHO 2012 report, Iran along with some of its neighboring countries (Azerbaijan and Turkey) are in the elimination phase of malaria control while prevention of re-introduction is the current phase in Oman and Iraq. Among other neighboring countries, Turkmenistan and Armenia are in malaria-free stage, and Afghanistan and Pakistan, with a long border with Iran, are malaria-endemic countries [2]. The entrance of foreigners into the country and travelling to the high-risk regions can lead to preservation of parasites reservoir, mainly in borderline areas [26].

Main technical interventions for control of malaria are based on reducing exposure to infective anopheles mosquito, chemotherapeutic measures, and prevention and control of malaria epidemics. It is clear that accurate diagnosis of malaria is the key point for disease management and also reduction of unnecessary use of antimalarial medicines [7, 27]. In this case, skilled personnel and method of diagnosis have important roles. Many studies have proved that polymerase chain reaction with high sensitivity and specificity has priority over traditional microscopy method for diagnosis of malaria [8, 9, 12, 28–30].

Since Fars province is situated in the local area of transmission of malaria [9], in the present study, we evaluated nested PCR for detecting plasmodia.

Sensitivity and specificity have been determined 95.7% and 97.9% for microscopy and 98% and 100% for PCR, respectively [27]. In another study, sensitivity and specificity of PCR were reported to be 100% versus 90.9% and 100% for microscopy [31].

Zakeri et al. have determined four negative samples by PCR as compared with thirteen negative cases by microscopy [12]. Also, Ebrahimzadeh et al. have obtained 11.55% PCR positive by extracting DNA from slides; it was undetectable by microscopy [9]. Microscopy assay showed lower sensitivity against PCR (37.3% versus 87.5%) for detection of *P. falciparum* [14].

Contrast with the above reports our results indicate a lower sensitivity for PCR in diagnosis of malaria. We just studied positive microscopic malaria samples and it should be better to do PCR using negative microscopic samples from suspected patients that refer to health care center as well as from positive cases. In this case, PCR power was evaluated in a similar and more suitable situation. Existence of different reports about the result of PCR based on extracted DNA from thick and/or thin blood smear (TBS) [9], previous investigations in SUMSPD [21], and routine molecular report in our department based on diagnosis of Leishmania encouraged us to extract plasmodia DNA from blood slides.

TBS has logistic advantages in comparison with whole blood including more reliability, easy storage for reasons of trace ability, and less difficulty to send the field study results for quality control [32, 33]. DNA from microscopic slides can be used in places where no facility exists for blood collection and storage (such as remote health stations), epidemiological studies and for diagnosis of the disease in travelers, retrospectively. Moreover, this method can be used for historical investigation in drug resistance or other genetic properties [9, 34].

Our slides were collected during two years and PCR was done after sample collection. Results of the present study showed that 132 (88.6%) out of 149 samples were positive by PCR after DNA extraction from TBS. Although our results are in contrast with our previous data on leishmaniasis investigation, in which all the microscopy positive slides were positive by PCR and 4 of the 14 microscopically negative smears were also PCR positive [21], these data show a higher positive percentage in comparison to other studies with a similar protocol (88.6 against 71% by Edoh et al.) [35]. Of course, the results were increased to 92% when DNA is extracted from thick films [35]. In the other study, although nested PCR using DNA from blood smears was positive in 62 out of 369 (16.8%) samples and this is the same portion observed microscopically, PCR has detected 22 malarial species that were not detectable by microscopy and vice versa [36]. Results of a study in Sistan-Baluchestan which has been performed on Giemsa-stained thin slide (some stored for more than one year) showed 11.55% PCR positivity in microscopic negative slides [9]. Different intrinsic and extrinsic factors can influence PCR performance on stained slides including quality of the reagents, condition of amplification, and conservation of the biological material used as a source of the isolation of DNA. Furthermore, some parasites may be lost during scrapping the slides [9, 36].

Some scientists believe that there is a relationship between parasitemia and PCR based on blood smear DNA extraction [36] whereas others have speculated that detection of plasmodia DNA extracted from archival slides is specific but its sensitivity is independent of parasitemia [37]. Parasitemia in the present study was evaluated for one third of the samples. The load of parasitemia varies from <0.1% to >5% in the slides.

Results in this study revealed no negative PCR cases among malarial samples obtained from Jask port. Jask samples had a very good quality (in case of shape, size of smear, and also staining condition) among the three sampling areas. We are also speculated that the process of cleaning additive materials (such as oil immersion) from the slides and method of DNA extraction may influence the quality of extracted DNA. However, such technique has successfully been used in the other comparable study in our country where all slides have been washed with ether and Na_2_HPO_4_ before DNA extraction [9].

The results of the present study revealed 7 (4.7%) mixed infections by microscopy and 22 (14.8%) by PCR. In other words, 10.1% mixed infection was detected by PCR more than microscopy.

Many previous investigations have detected high percentages of mixed cases in Iran. Nested PCR has detected 31 more mixed infections than microscopy [12]. Another molecular study using nested polymerase chain reaction has revealed 95 cases (29%) of both plasmodia [8]. Zakeri et al. in a study in Chahbahar have reported 33 more mixed plasmodia by PCR [13]. Although the comparative evaluation of malaria in Iran and its two endemic neighboring countries (Afghanistan and Pakistan) revealed that most cases were *P. vivax* by both molecular and microscopic assay, 6.5, 22, and 23.5% of mixed cases have respectively been detected by PCR in Afghanistan, Iran, and Pakistan [7].

*P. vivax* was the most prevalent species in the present investigation and this is consistent with the information from other studies in our country [7–9, 12–14, 18, 38]. According to our prevision based on available data from Center for Diseases Management and Control in Shiraz (unpublished data), most of malarial patients were foreign workers in Fars. In addition, existence of the majority of Iranian patients in Jask and Lengeh can confirm less endemic city in Fars in comparison with Hormozgan province.

## 5. Conclusions

Our results confirmed the considerable sensitivity of nested PCR for detection of the mixed infection cases that remain undiagnosed by microscopy. It is also concluded that the nested PCR is a suitable alternative method for reliable specific diagnosis of malaria species. Furthermore, DNA extraction from blood smear has different advantages including more reliability, easy storage, and lower need to shipment. Simultaneous application of PCR even based on TBS microscopy can facilitate access to the highest level of confidence in different areas. Protocols of sample preparation and DNA extraction may have important role in sensitivity of the molecular methods.

## Supplementary Material

The list of more essential instruments using in this research project are:
Electrophoresis tank and its supplements ( Biorad, USA), Thermocycler, refrigerated centrifuge, Gel documentation system(gel doc), Laminar flow hood (Class II), Hot plate stirrer, Balance Spectrophotometer and Incubator. A malaria research questionnaire were used for collecting all necessary data about the patients.

## Figures and Tables

**Table 1:** Frequency of malaria cases (*P. vivax*, *P. falciparum*, mixed infection and negative cases) based on microscopy and PCR in different studied areas.

Species	Method of detection	Detection of parasites from patient smears in different regions No. (%)			Total
		Shiraz Centre	Jask Centre	Lengeh Centre	
*P. vivax	PCR	69 (59.5)	17 (89.4)	9 (64.3)	95 (63.7)
	Microscopy	**97 (83.6)**	**18 (94.7)**	**11 (78.6)**	**126 (84.6)**

*P. falciparum	PCR	13 (11.2)	1 (5.3)	1 (7.1)	15 (10.1)
	Microscopy	**13 (11.2)**	**1 (5.3)**	**2 (14.3)**	**16 (10.7)**

Mixed infection	PCR	21 (18.1)	1 (5.3)	0 (0)	22 (14.8)
	Microscopy	**6 (5.2)**	**0 (0)**	**1 (7.1)**	**7 (4.7)**

Negative	PCR	13 (11.2)	0 (0)	4 (28.6)	17 (11.4)
	Microscopy	**0 (0%)**	**0 (0%)**	**0 (0%)**	**0 (0%)**

*Plasmodium.
