# Supplementary figures and images for: Molecular and Microscopic-Based Characterization of Plasmodium spp. in Fars and Hormozgan Provinces, South of Iran
Source: J Trop Med. 2014 Feb 6;2014:935469. doi: 10.1155/2014/935469 (PMC3988939; doi:10.1155/2014/935469)

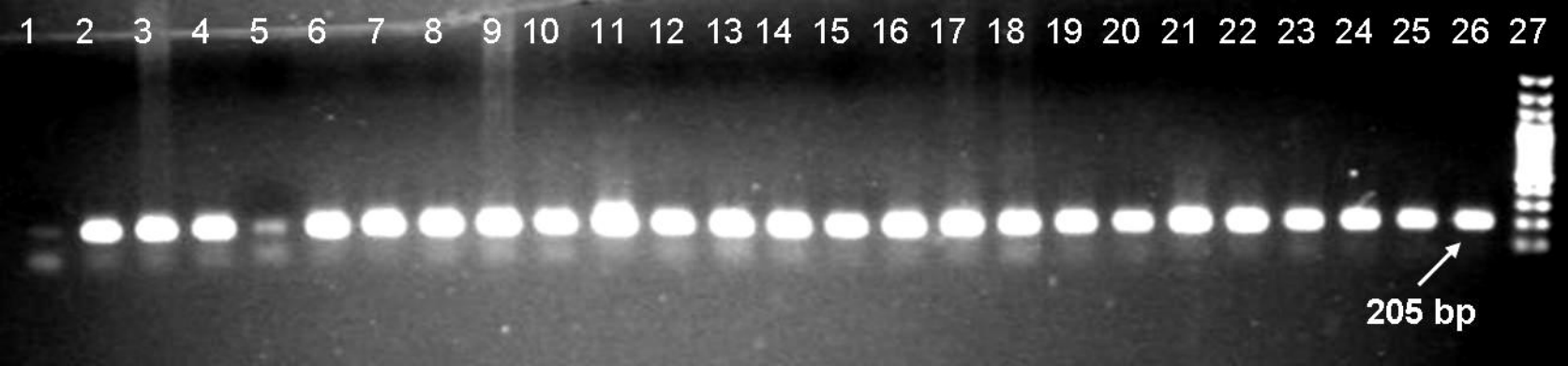

Supplement: Supplementary file 1 — The list of more essential instruments using in this research project are: Electrophoresis tank and its supplements ( Biorad, USA), Thermocycler, refrigerated centrifuge, Gel documentation system(gel doc), Laminar flow hood (Class II), Hot plate stirrer, Balance Spectrophotometer and Incubator. A malaria research questionnaire were used for collecting all necessary data about the patients. [file 935469.f1.pdf]
